# Case Report: An unexpected case of tumor regression in blue nevus melanoma following COVID-19 infection

**DOI:** 10.3389/fimmu.2025.1658609

**Published:** 2025-09-15

**Authors:** Yadriel Bracero, Emily Nghiem, Ajay K. Singh, Divya B. Kenchappa, Rachel Berglas, Shabnam Fidvi, Chaoyuan Kuang, Katia Papalezova, Beth McLellan, Bijal Amin, Yvonne M. Saenger

**Affiliations:** ^1^ Department of Oncology, Albert Einstein College of Medicine, Bronx, NY, United States; ^2^ Department of Surgery, Albert Einstein College of Medicine and Montefiore Medical Center, Bronx, NY, United States; ^3^ Department of Radiology, Albert Einstein College of Medicine, Bronx, NY, United States; ^4^ Department of Dermatology, Albert Einstein College of Medicine, Bronx, NY, United States; ^5^ Department of Pathology, Albert Einstein College of Medicine, Bronx, NY, United States

**Keywords:** blue nevus melanoma, tumor regression, GNA11 mutation, immunotherapy, immune checkpoint inhibitors (ICIs), COVID-19, case report

## Abstract

Blue nevus is a benign melanocytic lesion that appears as a blue or dark mole due to the presence of melanin deep within the skin. In rare cases melanoma can arise from this association with blue nevus, and entity termed blue nevus melanoma (BNM). BNM most frequently occurs on the scalp and is an aggressive subtype of melanoma which has the tendency to metastasize. Similar to acral melanoma, BNM has a distinct genetic profile, is less linked to sun exposure, and has an equal incidence in patients of European and non-European ancestry. It is also less responsive to immunotherapy. This case report describes a diagnosis of blue nevus-related scalp melanoma characterized by GNA11 mutation in a 50-year-old female Hispanic patient, with a tumor refractory to multiple courses of combination immunotherapy who developed metastases to the liver and underwent microwave ablation of the hepatic lesions. Her disease course was complicated by hospitalization for infection with coronavirus disease of 2019 (COVID-19) and autoimmune hepatitis. Months after being discharged, surveillance imaging revealed a decrease in size of the existing lesions without additional therapeutic intervention. While this unusual response can be attributed to multiple factors, this observation aligns with emerging reports suggesting potential tumor remission associated with COVID-19 infection.

## Introduction

Melanoma is less common than other types of skin cancer, yet it confers a significantly higher mortality rate (1). Treatment for cutaneous melanoma typically involves a combination of surgery, chemotherapy, radiotherapy, targeted therapy, and immunotherapy (2). Recently, the use of immunomodulatory agents has become a key component in the management of advanced melanoma. However, despite the introduction of several new immunotherapies, the prognosis for patients remains largely unfavorable (3). Since immunomodulation is a key element of advanced melanoma treatment, understanding the factors that influence immune response is critical for developing future treatment strategies and enhancing the understanding of the pathophysiology of advanced melanoma as effectiveness varies significantly across the different subtypes, specifically in rare variants such as blue nevus melanoma (BNM).

BNM is a rare and aggressive subtype of the melanoma arising from malignant transformation of cellular blue nevi benign melanocytic lesions defined by dermal proliferation of pigmented melanocytes. Fewer than 200 cases have been reported globally, highlighting its diagnostic and therapeutic challenges (4). Akin to uveal melanoma, BNM frequently harbors GNAQ and GNA11 mutations which drive a low tumor mutational burden (TMB) and create an immunosuppressive tumor microenvironment (TME) via the reduction of immune cell infiltration. These features likely contribute to their poor response to immune checkpoint inhibitors (ICIs), with response rates of about 10%, due to limited neoantigen availability and impaired T-cell activation (5). Reports have documented less efficacious responses to immunotherapy in BNM as compared to other subtypes of skin melanoma (6). Previous studies have suggested that the contraction of viruses such as coronavirus disease of 2019 (COVID-19) plays a role in spontaneous tumor regression in multiple different types of hematological cancers (7–10). While BNM’s poor ICI response is the focal point to the challenges of treating rare melanoma subtypes, emerging evidence suggests that systemic inflammatory response syndrome to COVID-19, may intrinsically heighten anti-tumor immune response. The present study reviews the case of a patient with BNM which progressed on ipilimumab, subsequently developing liver metastases treated with microwave ablation. This was followed by COVID-19 infection and concurrent autoimmune hepatitis. After months of unchanged surveillance imaging post ablation, imaging performed months after treatment of the hepatitis showed clinical regression, and the patient has been progression-free for 8 months. To the best of our knowledge, this is the first reported case of BNM reduction after COVID-19 infection.

## Case presentation

A 50-year-old Hispanic female from Guatemala was diagnosed with pT4a melanoma of the scalp in February 2021, for which a wide local excision (WLE) with left neck sentinel lymph node biopsy (SLNB) was performed. The Breslow thickness could not be determined but was reported as 9 mm without ulceration from an initial biopsy at an outside hospital. Using the Tumor-Node-Metastasis (TNM) system of cancer staging, pathology demonstrated a pT4aN2aM0, stage IIIC, BRAF wild-type, GNA11 mutated tumor with 3 of 13 lymph nodes showing micro-metastases. The risks and benefits of completion parotidectomy and neck dissection were weighed, and the decision was made to continue observation as completion dissection would have low likelihood of improving regional control. Adjuvant pembrolizumab (200 mg administered intravenously every 21 days) was initiated approximately one month postoperatively. After three infusions, treatment was complicated by stomatitis, sialadenitis, esophagitis, and candidiasis and was subsequently held while the patient completed a prednisone taper. One month later, given significant improvement of symptoms, adjuvant pembrolizumab was restarted. However, prior symptoms recurred after one infusion and the decision was made to discontinue treatment and perform imaging surveillance with computed tomography (CT) every three months.

In March 2023 the patient reported a new growth on her scalp near the surgical scar and punch biopsy confirmed recurrent melanoma with Breslow thickness of 4 mm without ulceration. Another WLE with SLNB was performed, with pathology demonstrating a pTxN3cM0 tumor (T stage not applicable as it was not possible to measure accurate thickness) with 0 of 5 lymph nodes showing evidence of melanoma. Despite the patient’s prior complications from pembrolizumab, the decision was made to begin adjuvant treatment due to disease recurrence, with a plan for one year of adjuvant immunotherapy. Having tolerated four infusions of pembrolizumab, therapy was then switched to nivolumab/relatlimab (480 mg nivolumab/160 mg relatlimab administered intravenously every 28 days, planned for 13 cycles) for a potentially broader immune response. However, following the fifth infusion, therapy was switched back to pembrolizumab as the patient presented to the emergency department with pain from oral sores causing difficulty with oral intake.

**Figure 1:**
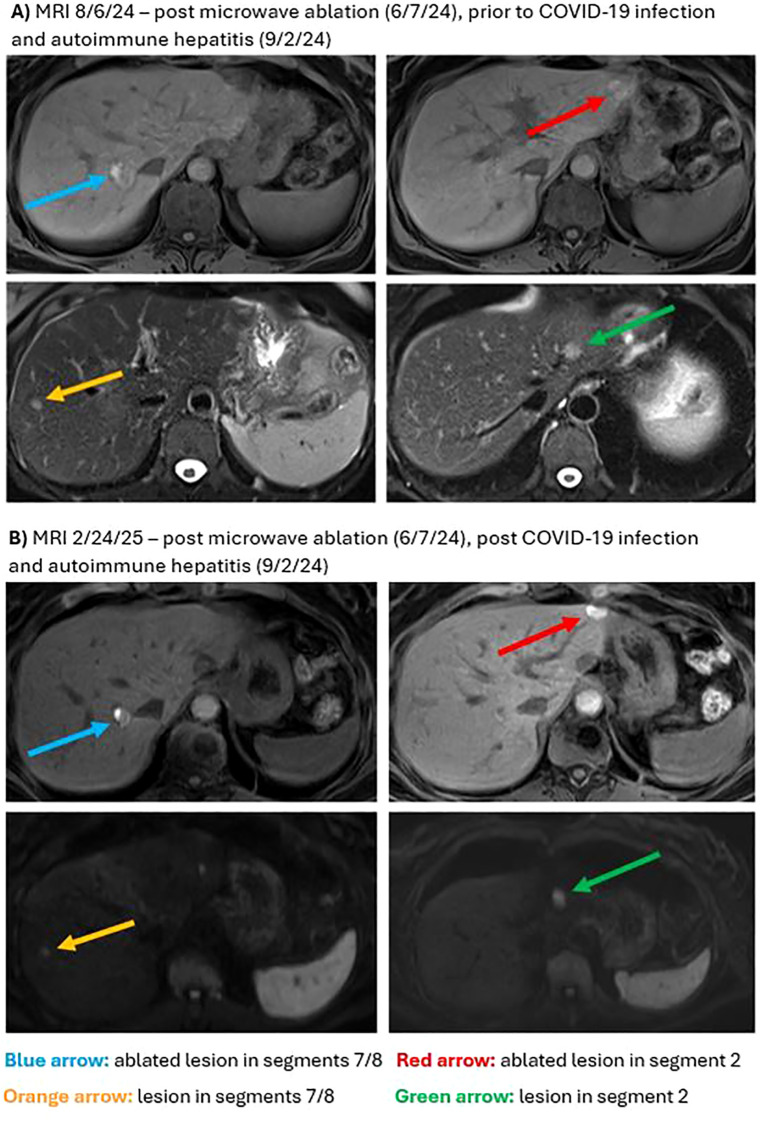
Magnetic resonance imaging (MRI) for hepatic metastases. **(A)** T1 weighted pre-contrast images from 8/6/24 demonstrate intralesional hyperintensity due to prior ablation in treated lesions in segments 7/8 (blue arrow) and in segment 2 (red arrow). T2 weighted images demonstrate hepatic metastases in segment 2 (green arrow) and in segments 7/8 (orange arrow). **(B)** Diffusion and T2 weighted MRI images from 2/24/2025 demonstrate unchanged hepatic metastases in segment 2 (green arrow) and in segments 7/8 (orange arrow). Ablated metastases in segments 7/8 (blue arrow) and in segment 2 (red arrow) are less conspicuous on T1 weighted pre-contrast images when compared to 8/6/24.

**Figure 2:**
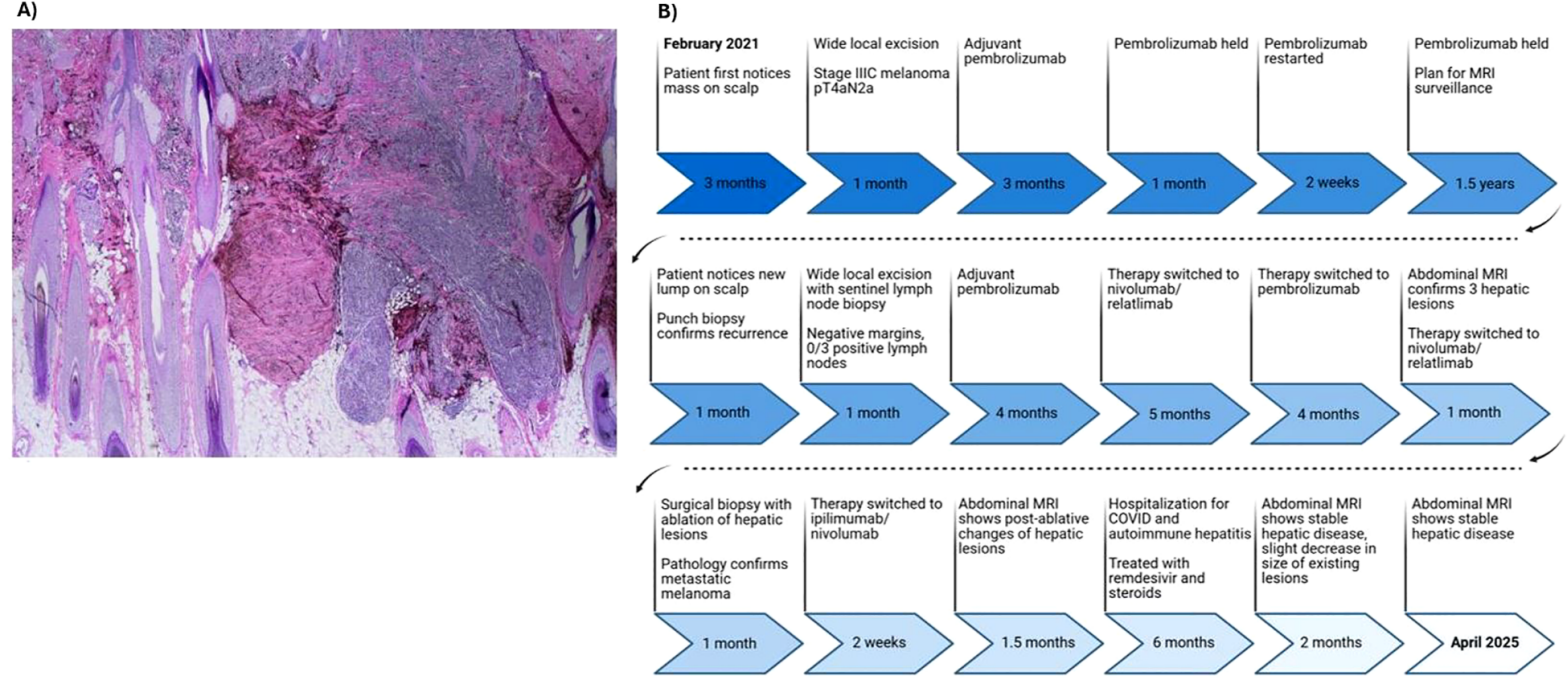
Histopathological examination and events timeline of blue nevus melanoma (BNM). **(A)** Hematoxylin and eosin stain (H&E) staining reveals characteristic features of malignant transformation forming a tumor mass. **(B)** Timeline of key events following initial diagnosis of blue nevus melanoma.

In March 2024, surveillance positron emission tomography (PET)/CT showed suspicious foci in the liver, which were confirmed shortly after by an abdominal magnetic resonance imaging (MRI) showing three hepatic lesions). Having then completed four infusions of pembrolizumab, adjuvant immunotherapy was again switched to nivolumab/relatlimab in light of the new liver lesions. In June 2024 the patient underwent surgical biopsy with microwave ablation (MWA) of lesions in liver segments II/III/VII. Liver biopsy pathology demonstrated neoplastic cells positive for melanocytic markers (S100, Melan A, HMB 45) consistent with metastatic melanoma. Postoperatively, adjuvant immunotherapy was switched to ipilimumab/nivolumab (3 mg/kg ipilimumab/1 mg/kg nivolumab administered intravenously every 21 days, planned for 4 cycles, followed by 240 mg nivolumab for one cycle). PET/CT and abdominal MRI performed after two doses of ipilimumab/nivolumab showed interval worsening of liver metastases which was difficult to interpret in the setting of recent ablation. Plans were made to refer the patient for tumor-infiltrating lymphocyte (TIL) therapy. Following three infusions of ipilimumab/nivolumab and approximately three weeks since her last infusion, the patient presented to the emergency department due to worsening cough, myalgias, and fever of 102.6 °F (39.2 °C). She was found to be COVID-19 positive, with RT-qPCR results showing a cycle threshold (Ct) value of 32.31. Laboratory tests were remarkable for elevated liver enzymes, including aspartate transaminase (AST) 1037 U/L (reference range <37 U/L), alanine transaminase (ALT) 1245 U/L (reference range <42 U/L), and alkaline phosphatase (ALP) 255 U/L (reference range <130 U/L). A viral hepatitis panel resulted as negative, and hepatology recommended liver biopsy to confirm suspected immune-mediated hepatitis. Given the patient’s reservations to undergoing an invasive procedure, the decision was made to start a trial of steroids and monitor for improvement in liver enzymes. She was treated with remdesivir for COVID-19 as well methylprednisolone for presumed checkpoint inhibitor-mediated autoimmune hepatitis. Though still elevated, liver enzymes showed marked improvement after initiation of steroids with AST 229 U/L, ALT 762 U/L, and ALP relatively stable at 274 U/L. On hospital day 10 after symptomatic improvement, the patient was discharged with a steroid and mycophenolate mofetil (MMF) taper. Adjuvant immunotherapy was held due to continually elevated liver enzymes. Surveillance imaging performed five months post hepatic ablation showed no changes when compared to imaging performed two months post hepatic ablation (**Supplementary Figure 1**). Nine months post hepatic ablation, surveillance imaging showed stable disease in the liver with no new lesions and a slight decrease in size of multiple existing lesions. Using RECIST 1.1 scoring, three target lesions showed 25% tumor shrinkage and two non-target lesions showed no progression, representing a clinical regression of the tumor and stable disease per RECIST 1.1 guidelines. Representative MRI images from before and after COVID-19 infection and autoimmune hepatitis are presented in **Figures 1A, B**. A histopathological image of a biopsy of the BNM with a comprehensive overview of the patient history and treatment timeline is presented in **Figures 2A, B**. As of late 2024 the patient has remained off immunotherapy with recommendations from multidisciplinary tumor board to continue observation in the setting of stable imaging.

## Discussion and conclusion

BNM represents a rare but aggressive variant of melanoma that poses significant diagnostic and therapeutic challenges due to similarity with uveal melanoma. While benign blue nevi exist in three primary forms (common, cellular, and combined), BNM is distinguished by its malignant behavior and poor prognosis (11). Similar to uveal melanoma, BNM frequently harbors GNAQ and GNA11 mutations, which drive a low TMB and create an immunosuppressive microenvironment, contributing to its limited response to ICIs. The present case highlights a patient with BNM who experienced tumor regression following COVID-19 infection despite prior progression on multiple lines of immunotherapy. This unexpected response raises intriguing questions about the interplay between viral infections and tumor biology, particularly in malignancies without strong response to initial treatment with checkpoint blockade.

Although melanomas can periodically respond to reinduction with checkpoint blockade therapy, it is rare for patients to achieve a response to ipilimumab or nivolumab after failing multiple courses of pembrolizumab and/or nivolumab/relatlimab (12–14). It is important to note that microwave ablation of the hepatic lesions represents a confounding treatment factor, although imaging performed five months post ablation still had not demonstrated a decrease in size of the lesions (**Supplementary Figure 1**). It is possible that the combination of ipilimumab/nivolumab with both hepatic ablation and COVID-19 together induced the episode of autoimmune hepatitis with subsequent tumor response. An option for BNM is Tebentafusp, a recently approved drug for occult melanoma specifically targeting the gp100 protein that is overexpressed in GNA11 mutant tumors (5). While Tebentasup offers a more targeted approach for GNA11 mutant melanomas which have previously been unaffected by ICI, it is restricted to patients who express the HLA-02 haplotype, one which is most common in patients of European ancestry.

Few cases exist in the literature reporting spontaneous tumor regression in various malignancies following COVID-19 infection. One study described a metastatic colorectal cancer patient undergoing unexpected tumor reduction after COVID-19 infection, supporting the hypothesis that viral infections can enhance anti-tumor immunity (15). Another study suggested that COVID-19 could trigger an immune response leading to hematological malignancy remission (9). Still, the relevance of these findings associated with BNM remains unclear since this subtype of melanoma is typically resistant to immune checkpoint blockade (16) However, the innate immune activation triggered by COVID-19 could demonstrate an alternate mechanism for tumor suppression by circumnavigating the traditional immune evasion strategies employed by GNA11 driven cancers. Studies have theorized that this is due to the COVID-19 infection potentially evoking an anti-tumor response through cross-presentation of tumor antigens in the setting of inflammatory mediators produced by the infectious agent, similar to mechanisms proposed for oncolytic viral therapy or even manipulation of the microbiome. Alternatively, anti-tumor immunity can be evoked by non-specific activation of natural killer cells stimulated by the inflammatory cytokines produced in response to COVID-19 infection (10). As the immunosuppressive TME created by GNA11 mutations is largely in part due to dampened T-cell response, the cytokine storm and innate immune activation associated with COVID-19 infection could prove especially efficacious in the setting of BNM (5). As demonstrated in this case report, the patient presented with cutaneous melanoma with liver metastases, a common site of melanoma metastases, which improved after a series of events including hepatic ablation, contraction of COVID-19, and autoimmune hepatitis (17). Although confounding factors exist in this case, it echoes a similar previously reported case in which spontaneous tumor regression of colon cancer with liver metastases occurred in a patient who was undergoing immunotherapy, but only demonstrated regression after contracting COVID-19 (15).

This study is limited in that it describes the experience of a single patient, and as such, the findings may not be generalizable to all patients with melanoma who develop COVID-19. The history of hepatic ablation performed three months prior to COVID-19 infection also makes it difficult to discern to what extent various factors contributed to tumor response. Additionally, the observed tumor response has only been associated with COVID-19 infection, emphasizing the need for exploration in the setting of other viral infections. In conclusion, this case presents a unique instance of tumor regression in BNM after COVID-19 infection, a phenomenon not previously reported. Although the precise mechanism remains unclear and may be multifactorial, the clinical symptoms and diagnostic findings suggest that COVID-19 infection can induce immunomodulatory changes that contribute to enhanced anti-tumor response. It remains possible that intermittent viral infections may modulate both response and toxicities from immunotherapy.

## Data Availability

The original contributions presented in the study are included in the article/**Supplementary Material.** Further inquiries can be directed to the corresponding author.

## Supplementary material

Supplementary Figure 1 Magnetic resonance imaging (MRI) for hepatic metastases. Diffusion weighted MRI images from 11/15/2024 demonstrate unchanged hepatic metastases in segments 7/8 (blue arrow), segment 2 (red arrow), segment 2 (green arrow), and segments 7/8 (orange arrow) when compared to 8/6/24.

Supplementary Table 1 Comprehensive timeline of case presentation.
