# Supplementary figures and images for: Case Report: An unexpected case of tumor regression in blue nevus melanoma following COVID-19 infection
Source: Front Immunol. 2025 Sep 15;16:1658609. doi: 10.3389/fimmu.2025.1658609 (PMC12477163; doi:10.3389/fimmu.2025.1658609)

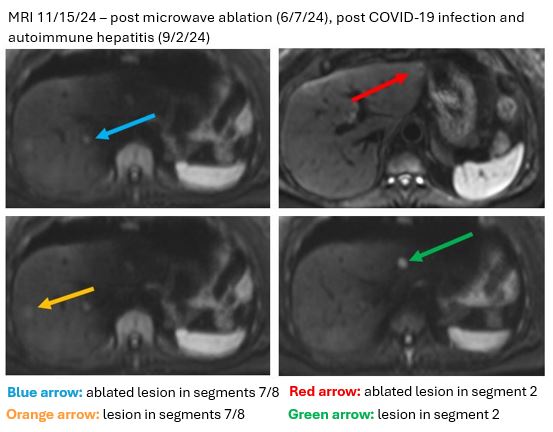

Supplement: Supplementary Figure 1 — Magnetic resonance imaging (MRI) for hepatic metastases. Diffusion weighted MRI images from 11/15/2024 demonstrate unchanged hepatic metastases in segments 7/8 (blue arrow), segment 2 (red arrow), segment 2 (green arrow), and segments 7/8 (orange arrow) when compared to 8/6/24. [file Image1.jpg]

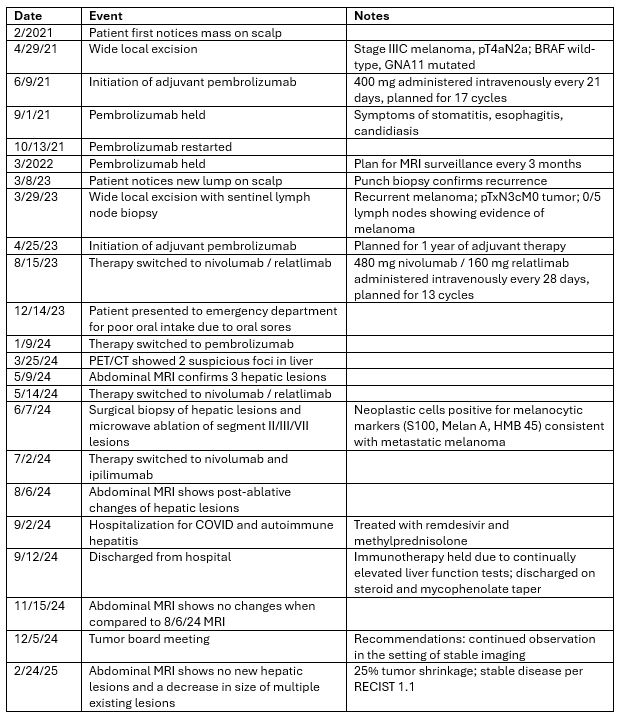

Supplement: Supplementary Table 1 — Comprehensive timeline of case presentation. [file Image2.jpg]
